# Global Survey and Expressions of the Phosphate Transporter Gene Families in *Brassica napus* and Their Roles in Phosphorus Response

**DOI:** 10.3390/ijms21051752

**Published:** 2020-03-04

**Authors:** Jin Yang, Jie Zhou, Hong-Jun Zhou, Mang-Mang Wang, Ming-Ming Liu, Yun-Zhuo Ke, Peng-Feng Li, Jia-Na Li, Hai Du

**Affiliations:** 1 College of Agronomy and Biotechnology, Southwest University, Chongqing 400716, China; yangjin9963@163.com (J.Y.); zj893422105@163.com (J.Z.); hsymjj@email.swu.edu.cn (H.-J.Z.); mangmangwang16@126.com (M.-M.W.); mingmingliu95@163.com (M.-M.L.); kyz2014@email.swu.edu.cn (Y.-Z.K.); pengfengli17@126.com (P.-F.L.);; 2 Academy of Agricultural Sciences, Southwest University, Chongqing 400716, China

**Keywords:** *Brassica napus* L., PHT gene family, phylogenetic analysis, expression analysis

## Abstract

Phosphate (Pi) transporters play critical roles in Pi acquisition and homeostasis. However, currently little is known about these genes in oil crops. In this study, we aimed to characterize the five Pi transporter gene families (PHT1-5) in allotetraploid *Brassica napus*. We identified and characterized 81 putative PHT genes in *B. napus* (*BnaPHTs*), including 45 genes in PHT1 family (*BnaPHT1s*), four *BnaPHT2s*, 10 *BnaPHT3s*, 13 *BnaPHT4s* and nine *BnaPHT5s*. Phylogenetic analyses showed that the largest PHT1 family could be divided into two groups (Group I and II), while PHT4 may be classified into five, Groups I-V. Gene structure analysis revealed that the exon-intron pattern was conservative within the same family or group. The sequence characteristics of these five families were quite different, which may contribute to their functional divergence. Transcription factor (TF) binding network analyses identified many potential TF binding sites in the promoter regions of candidates, implying their possible regulating patterns. Collinearity analysis demonstrated that most *BnaPHTs* were derived from an allopolyploidization event (~40.7%) between *Brassica rapa* and *Brassica oleracea* ancestors, and small-scale segmental duplication events (~39.5%) in the descendant. RNA-Seq analyses proved that many *BnaPHTs* were preferentially expressed in leaf and flower tissues. The expression profiles of most colinearity-pairs in *B. napus* are highly correlated, implying functional redundancy, while a few pairs may have undergone neo-functionalization or sub-functionalization during evolution. The expression levels of many *BnaPHTs* tend to be up-regulated by different hormones inductions, especially for IAA, ABA and 6-BA treatments. qRT-PCR assay demonstrated that six *BnaPHT1*s (*BnaPHT1.11*, *BnaPHT1.14*, *BnaPHT1.20*, *BnaPHT1.35*, *BnaPHT1.41*, *BnaPHT1.44*) were significantly up-regulated under low- and/or rich- Pi conditions in *B. napus* roots. This work analyzes the evolution and expression of the PHT family in *Brassica napus*, which will help further research on their role in Pi transport.

## 1. Introduction

Phosphorus (Pi) plays a central role in energy metabolism, signal transduction cascades, regulation of enzymes and as a structural element in nucleic acids and phospholipids [1]. Despite being one of the most abundant macronutrients in plant tissues, Pi forms organic complexes and undergoes inorganic fixation with cations in soil solution [2]. It was widely proved that Pi is very important for crop growth and the crop yield can be obviously increased by applying phosphate fertilizer [3]. However, Pi is one of the least available plant macronutrients [4]. Therefore, it is crucial to increase the efficiency of Pi absorption and utilization in crop production.

In order to improve Pi uptake efficiency from the soil, plants have developed an array of morphophysiological, biochemical, and molecular adaptations to adapt to the low availability of Pi [2,5,6], including reduced plant growth, changed root morphology and architecture, and expressed Pi response genes [7,8,9,10,11]. Among the molecular response processes, the Pi transporters play a pivotal role in the acquisition and mobilization of Pi in plants [8,12,13,14]. To date, five Pi transporter families have been identified and isolated in plants: PHT1-5 [15,16]. Among them, the PHT1 family is the high-affinity Pi transporter which represents a group of Pi carriers usually found in plasma membrane [17]. Members of this family play a wide role in Pi uptake and remobilization throughout plant development. For example, *Arabidopsis AtPHT1;1* and *AtPHT1;4* play major roles in Pi acquisition in both low- and high- Pi environments [12,13,14]; rice *OsPht1;1* [18], *OsPht1;2* and *OsPht1;6* [19] were involved in Pi uptake and translocation. The other four families (PHT2-5) also play important roles in plant Pi transport. For example, *Arabidopsis AtPHT2;1* in PHT2 family is a chloroplast low-affinity Pi transporter [20,21]; the PHT3 homologs act as a Pi/H+ symporter Pi/OH^−^ antiport to play a critical role in Pi exchange between cytoplasm and mitochondria matrix [22,23]; *AtPHT4;6* in PHT4 family transports Pi out of the Golgi lumenal space to be recycled after release from glycosylation [24]; while the PHT5 family members are known to be vacuolar Pi transporter [16]. Therefore, members of the five PHT families play critical roles in plant Pi uptake, translocation and mobilization. Based on the increasing available sequenced genome datasets, the PHT gene families were globally identified and analyzed in many plant species, such as *Arabidopsis* [25], rice [26], and poplar [27]. However, most of these studies merely focus on the PHT1 family, and few reports about the whole PHT family at genome-wide level are available.

*Brassica napus* L. is an important oil crop with a world production of over 60 million tons each year. It is demonstrated that *B. napus* is sensitive to Pi deficiency. A lack of available Pi in soil may inhibit its growth and even its yield and quality [28]. Although the PHT genes (*PHTs*) are important for plant Pi uptake and transport, only the PHT1 family [29] and one gene (*BnPHT1;4*) [30] were studied in *B. napus* to date. Global identification, and systematic evolution and expression profile analysis of the PHT families’ genes in *B. napus* genome will provide fundamental information for further functional assays of their roles in Pi uptake and translocation in this species.

In the present study, we applied a systematic identification and classification of the five PHT gene families in *B. napus* genome. The physicochemical properties, subcellular localization, gene structure, phylogenetic relationship, and evolution mechanism of the candidate *PHTs* in *B. napus* genome (*BnaPHTs*) were analyzed by diverse Bioinformatic methods. The spatiotemporal expression profiles of the candidates in 50 *B. napus* tissues and/or organs across different developmental stages were analyzed, and we found that most genes were preferentially expressed in leaf and flower tissues. Moreover, many *BnaPHTs* were proved to have hormone-induced expression profiles (IAA, auxin; GA_3_, gibberellin; 6-BA, cytokinin; ABA, abscisic acid and ACC, ethylene), based on our RNA-Seq dataset. In addition, their expression patterns in low- or high- Pi stresses were further assessed by qRT-PCR method, which proved that several genes in PHT1 family are significantly up-regulated under low- Pi conditions in *B. napus* roots.

## 2. Results

### 2.1. Identification of PHT Genes in B. napus

To identify the *PHTs* in the *B. napus* genome, a preliminary BLASTP search was performed using the sequences of known *Arabidopsis* PHT proteins (AtPHTs) as queries, based on the available *B. napus* genome database in GENOSCOPE (Darmor–*bzh*, http://www.genoscope.cns.fr/brassicanapus/) [31]. In each case, a large number of deduced PHT homologous sequences were acquired. The redundant sequences were firstly discarded from our dataset. Then the remaining sequences were verified by SMART (http://smart.embl-heidelberg.de/). The sequences possessing incomplete open reading frames (ORFs), especially long deletion were excluded from further analysis, such as BnaCnng51590D (Table S1). Finally, we obtained 81 typical PHT genes with relative complete ORFs in *B. napus* (*BnaPHTs*), and temporarily named them according to their order on the corresponding chromosomes (Table S1). To our knowledge, the candidate *PHT* genes in *B. napus* consist of the largest PHT family known to date [25,26,27,32]. This may attribute to *B. napus* (AACC. n=19) is an allotetraploid produced by the recent hybridization between *B. rapa* (AA. *n*=10) and *B. oleracea* (CC. *n*=9) about 7500 years ago.

Similarly, we also identified 46 candidate PHT homologs in *Brassica rapa* (*BrPHTs*) and 26 PHT homologs in *Brassica oleracea* (*BoPHTs*) from Phytozome v12 (https://phytozome.jgi.doe.gov/) by the same method (Table S2).

### 2.2. Phylogenetic Analysis of B. napus PHT Gene Family

To determine the evolutionary relationship of *B. napus* PHT gene families, we constructed a Neighbor-Joining (NJ) tree and a Maximum Likelihood (ML) trees of the candidate 165 PHT proteins from *B. napus* (80), *Arabidopsis* (22), *B. rapa* (42) and *B. oleracea* (21) based on the alignment of the full-length amino acid sequences using MEGA5.0 respectively. Five members (BnaPHT1.24, BrPHT1.25, BrPHT5.3, BoPHT1.4 and BoPHT1.5) were excluded from the phylogenetic trees because of technological reason (lacking common sites for the sequence pairs due to large sequence deletion).

**Figure 1:**
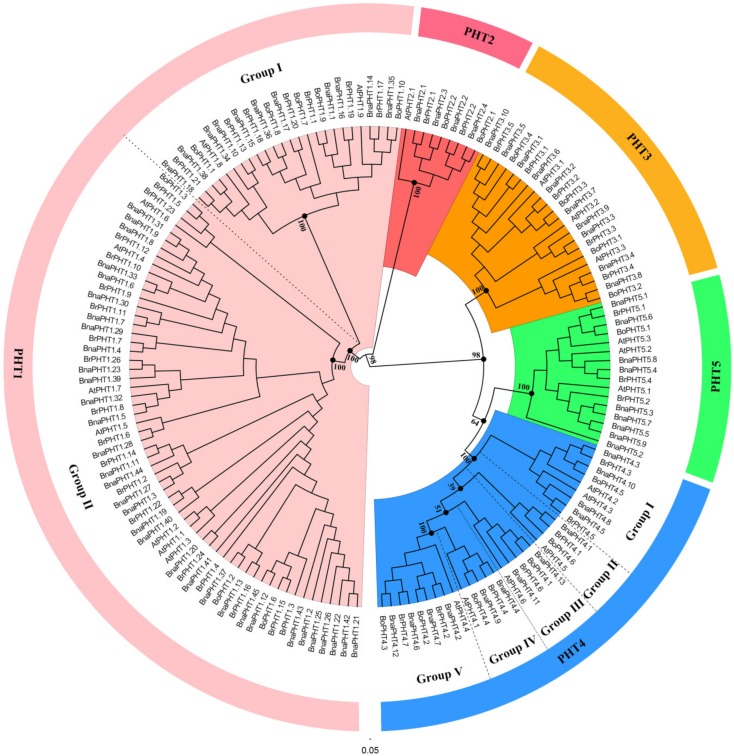
Phylogenetic analysis of phosphate transporter (PHT) gene families in *Brassica napus*, *Arabidopsis*, *Brassica rapa* and *Brassica oleracea*. The colored background indicates different PHT family. The different groups within the PHT1 and 4 families were separated by dashed lines, respectively.

The topologies and bootstrap support values of the NJ and ML trees were highly congruent (Figure 1 and Figure S1), and the candidate PHT members were clustered into five main clusters: PHT1-5 families (Figure 1). The PHT1 family is the largest one which includes nine genes in *Arabidopsis* (*AtPHT1s*), 25 in *B. rapa* (*BrPHT1s*), 8 *B. oleracea* (*BoPHT1s*) and 44 in *B. napus* (*BnaPHT1s*); the PHT2 family contained one *AtPHT2* gene, two *BrPHT2s*, two *BoPHT2s* and four *BnaPHT2s*; the PHT3 family included three *AtPHT3s*, five *BrPHT3s*, four *BoPHT3s* and 10 *BnaPHT3s*; the PHT4 family was consisted of six *AtPHT4s*, seven *BrPHT4s*, six *BoPHT4s* and 13 *BnaPHT4s*; while the PHT5 family contained three *AtPHT5s*, three *BrPHT5s*, one *BoPHT5* gene and nine *BnaPHT5s* (Figure 1). Consistent with previous studies [33], the PHT1 family could be further classified into two groups, where group I composed of 11 *BnaPHT1s*, seven *BrPHT1s* and five *BoPHT1s* that were homologous to *AtPHT1.8* and *AtPHT1.9*; group II contained 33 *BnaPHT1s*, 18 *BrPHT1s* and three *BoPHT1s* that were homologous to *AtPHT1.1*-*AtPHT1.7*. Similarly, the PHT4 family might be classified into five groups (Group I-Ⅴ) (Figure 1), where group I contained nine Brassica members and *AtPHT4.2* and *AtPHT4.3*; group II consisted of *AtPHT4.5* and Brassica homologs; group III included *AtPHT4.6* and four Brassica homologs; group IV composed of *AtPHT4.1* and four Brassica homologs, and group Ⅴ included *AtPHT4.4* and eight Brassica homologs.

Physicochemical property analysis showed that the molecular weight (MW) of candidate BnaPHT proteins (BnaPHTs) ranged from19.20-78.90 kDa, the isoelectric point (IP) is 5.44–10.01 (Table S1). The amino acid length of BnaPHTs varied from 279 to 704 aa, and these of PHT5 family are almost the longest while the PHT3 family are obviously shorter than the others. Subcellular localization analysis found that members of PHT1 and 4 families are located on the cell membrane, and members of PHT2 and 3 families are located on chloroplast and mitochondrion, respectively. In contrast, the majority of the PHT5 family are located on the cell membrane and vacuole except for one (*BnaPHT5.2)*, which was located on the cell membrane and chloroplast. The diverse subcellular localization features (Table S1) of these five families were highly consistent with the functional diversity of their homologs in plants [33].

### 2.3. Gene Structure of PHT Genes

**Figure 2:**
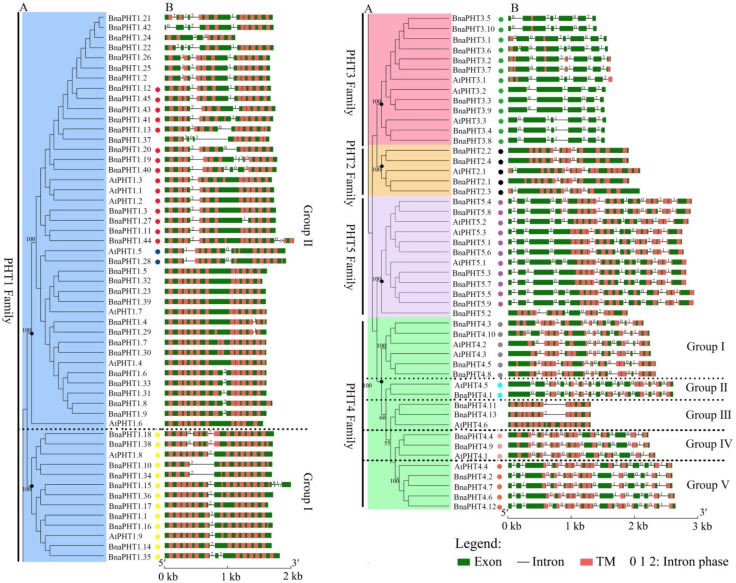
Gene structures of candidate PHT genes (*PHTs*) across different families. (**A**) Phylogenetic analysis of PHT families in *B. napus* and *Arabidopsis*. Colored background indicates genes belong to different families. The dashed lines represent the genes belong to different groups. (**B**) Gene structure of *PHTs* in *B. napus* (*BnaPHTs*) and *Arabidopsis* (*AtPHTs*). Exon was indicated by green boxes, transmembrane (TM) by red boxes, and the spaces between the colored boxes correspond to introns. Numbers 0, 1, 2 represent introns in phases 0, 1, and 2, respectively. The colored dots represent the conservative intron insertion patterns corresponding to the Figure S2, respectively.

To gain insight into the structural feature of *PHTs* in *B. napus*, *Arabidopsis*, *B. rapa* and *B. oleracea*, we compared their full-length cDNA and genomic DNA sequences to determine the insertion site and number of introns within each gene. The results of *B. napus* and *Arabidopsis PHTs* were viewed by GSDS software in Figure 2. The intron patterns of the five PHT families across these four species were presented in a schematic form in Figure S2, respectively.

As shown in Figure 2, the coding regions of most *PHTs* in *B. napus* and *Arabidopsis* are disrupted by introns varying from one to 14, except for 9 members in PHT1 family (Figure 2B). Among the five PHT families, the gene structures in PHT2, 3, and 5 families were highly conserved in terms of the exon-intron structure with only a few exceptions that may be attributed to low genome sequence quality, respectively (Figure 2B). The genes in PHT2 family possessed 2-3 introns, those in PHT3 family possessed 4-6 introns; and those in PHT5 family generally possessed 8-9 introns except for *BnaPHT5.2*, *BrPHT5.2* and *BrPHT5.3* (containing four or six introns). In contrast, the gene structures in PHT1 and 4 families were relatively less conserved. The *PHTs* in PHT1 family generally possessed 1-4 introns, where 21 members of the 54 *PHT1s* have one intron, 14 *PHT1s* contain two introns, ten *PHT1s* have three or four introns, respectively (Figure 2B). However, the gene structures were generally conserved in the same branch in PHT1 family that was consistent with their group classification in the phylogenetic tree, particularly the group I (Figure 2A), forming three conserved intron patterns across the four species investigated (Figure 2B). Similarly, the intron patterns of *PHTs* in PHT4 family could be summarized into -five conserved intron patterns (Figure 2B). Similar results were observed in *B. rapa* and *B. oleracea PHTs* (Figure S2). These results indicated that the gain and loss of introns likely occurred in these two families during evolution.

Taken together, our results showed that the exon-intron patterns were generally conserved in each family or group in these five PHT families across the four species, indicating that each pattern shares a common ancestor.

### 2.4. Sequence Analysis of B. napus PHT Proteins

**Figure 3:**
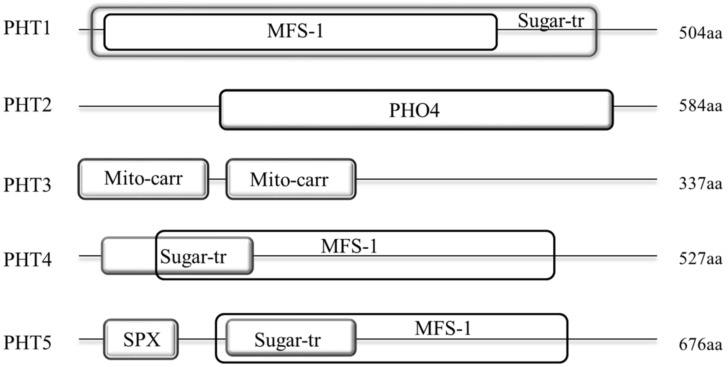
Architecture of conserved protein domains in the five PHT families in *B. napus*. The conserved domains by Simple modular architecture research tool (SMART) were represented by different size boxes. The average protein length of each family is indicated on the right.

To analyze the sequence features of different PHT families in *B. napus*, we predicted the domains in the full-length protein sequences of the five PHT families using SMART software (Figure 3 and Table S3).

Our results showed that the protein structures of these five families were generally different. Among them, members of PHT1, 4 and 5 families display a similar domain architecture that they all contain the major facilitator superfamily (MFS-1, functions in transmembrane transport) and Sugar (and other) transporter (Sugar-tr, functions in transmembrane transport) domains. However, the sequence features of these two domains were not conservative among these three families with the MFS-1 and Sugar-tr domains are partial or even completely overlapped in these families. As shown in Figure 3, the MFS-1 domain (~400 aa) in PHT1 family is completely overlapped by the Sugar-tr domain (~480 aa) near the N-terminus. In contrast, the Sugar-tr domain is completely overlapped by the MFS-1 domain in PHT5 family (Figure 3). In addition, nearly all BnaPHT5s (except BnaPHT5.2) contained an additional highly conservative SYG1/Pho81/XPR1 (SPX, functions in phosphate transport) domain before the MFS-1 domain with a length of approximately 52 aa. In contrast, the MFS-1 and Sugar-tr domains are partial overlapped in PHT4 family. Furthermore, compared to PHT1 family, the MFS-1 domains were relatively highly conserved in both of PHT4 or PHT5 families, in contrast, the Sugar-tr domains were relatively poorly conserved in these two families. However, the sequence features of PHT2 and PHT3 were quite different from the PHT1, 4 and 5 families (Figure 3). The average lengths of the BnaPHT2s and BnaPHT3s were 584 aa and 337 aa, respectively. Moreover, the BnaPHT2s contained the highly conserved (PHO4, functions in inorganic phosphate transmembrane transport) domain near the C-terminus (approximately 400 aa in length), whereas the BnaPHT3s included two separated conservative Mitochondrial carrier (Mito-carr, substrate carrier proteins that are involved in energy transfer) domains at the N-terminus (about 90 aa for each domain).

The TMPRED software analyses showed that nearly all members of these five PHT families contained different numbers of transmembrane (TM) domains, suggesting this domain is important for their functions in plants. As shown in Figure 2B, all the BnaPHT1s contained 6-14 transmembrane (TM) domains; the BnaPHT2s contained 10-12 TM domains which are consistent with the previous report [5,20]; the BnaPHT4s and BnaPHT5s contained 8-14 and 11-13 TM domains, respectively; in addition, most of the BnaPHT3s possessed only 1-5 TM domains, but four members (BnaPHT3.3, BnaPHT3.4, BnaPHT3.8 and BnaPHT3.9) which belonged to the same branch with AtPHT3.2, and AtPHT3.2 lacked the TM domains. Because all the BnaPHT3s contained two mito-carr domains (including BnaPHT3.3, BnaPHT3.4, BnaPHT3.8 and BnaPHT3.9), the PHT3 family may rely on the mito-carr domain instead of the TM domain to function as Pi transporters. Furthermore, the sequence characteristics and spatial positions of the TM domains are generally conserved in each family or group. However, their locations were different across different families, such as those of BnaPHT5s were located at the C-terminal while those in BnaPHT1s were distributed in the full length ORF region.

Taken together, our results indicate that the sequence characteristics were conserved in each of the five PHT families or groups but were different across them, which may have contributed to functional divergence across different families.

### 2.5. Chromosomal Distribution and Duplication of BnaPHTs

To investigate the gene duplication events within *B. napus* PHT families, we analyzed the chromosomal locations and collinearity relationship of candidates based on the annotion information in Genoscope database.

**Figure 4:**
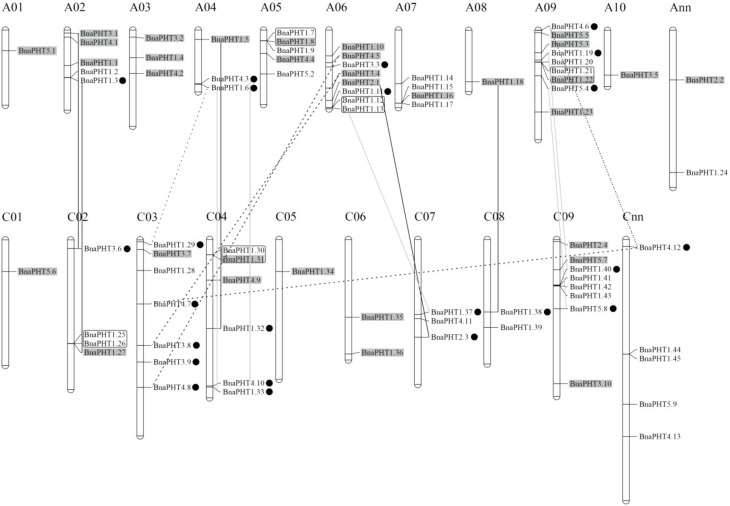
Distribution of *PHT* genes on *B. napus* chromosomes. The 81 *BnaPHTs* were mapped on the 19 chromosomes, except for seven genes. The Ann and Cnn chromosomes represent the chromosomal fragments mapped to A_n_ or C_n_ subgenome respectively, but the locations of these are as yet unclear. The genes in the gray background were originated from the whole genome duplication event (WGD). The black, gray and dashed lines represent the genes involved in homologous exchange (HE), segmental exchange (SE), and segmental duplication (SD) events with the new duplicated genes are marked in black dot, respectively. The genes in the black box represent tandem duplication (TD) pairs.

Our results showed that the candidate *BnaPHTs* were distributed on all the 19 *B. napus* chromosomes, unevenly (Figure 4 and Table S1). The numbers of *BnaPHTs* between the two subgenomes are uneven, where the A_n_-subgenome has 40 *BnaPHTs* while the C_n_-subgenome possess 34 *BnaPHTs*. The genes distribution on each chromosome was bias, such as the A09 had a maximum number of nine genes, while A01 and A08 had a minimum of one gene. In addition, the chromosome information of seven genes was currently unknown (Table S1).

On the basis of the collinearity analyses between *B. napus*, *B. rape* and/or *B. oleracea*, we found that 65 of the 81 *BnaPHTs* have colinear relationships with *BrPHTs*, *BoPHTs* and/or *BnaPHTs* (Table S5). Of these, ~50.8% of the colinear genes were inherited from *B. rapa* (22 genes, ~33.8%) or *B. oleracea* (11 genes, ~17.0%) genomes. Given that *B. napus* is a new allotetraploid that was hybridized by *B. rapa* and *B. oleracea* about 7500 years ago, these results demonstrated that most of the *BnaPHTs* (33/81, ~40.7%) were derived from its parents through allopolyploidy. Moreover, up to ~18.5% *BnaPHTs* were demonstrated to be derived from the segmental exchange (SE) event; ~7.4 % genes were from the homologous exchange (HE) event; and ~7.4% genes were from the segmental duplication (SD) event (Table S5), indicating this family tend to undergo small-scale duplication events in *B. napus* genome. Furthermore, we found that all the five PHT families have the largest number of genes that are orthologous pairs between *B. napus* and *B. rapa* or *B. oleracea*, including 14 pairs in PHT1 family, three pairs in PHT2 family, six pairs in PHT3 family, and five pairs in both of PHT4 and 5 families (Table S5). There are three, three and two pairs of *BnaPHT1s* involved in HE, SE, and SD events respectively; one pair of PHT2-4 families in HE event respectively; one pair of PHT3-5 families in SE event respectively; and one pair of PHT3 family, and three pairs of PHT4 family in SD event respectively (Table S5). In addition, five pairs of tandem duplication (TD) genes (*BnaPHT1.7/1.8*, *BnaPHT1.12/1.13*, *BnaPHT1.21/1.22*, *BnaPHT1.25/1.26*, and *BnaPHT1.30/1.31*) were identified (~6.2%), according to their distributions on chromosomes and sequence similarity (Table S5).

Overall, our results demonstrated that allopolyploid (large-scale duplication event) and small-scale duplication events after genomic heterogeneous doubling (HE, SE, and SD) were the main driving force for the large expansion of PHT gene family in *B. napus* genome. However, the trends of small-scale duplication events in the five PHT families were different.

### 2.6. Transcription Factor Binding Network Analysis

**Figure 5:**
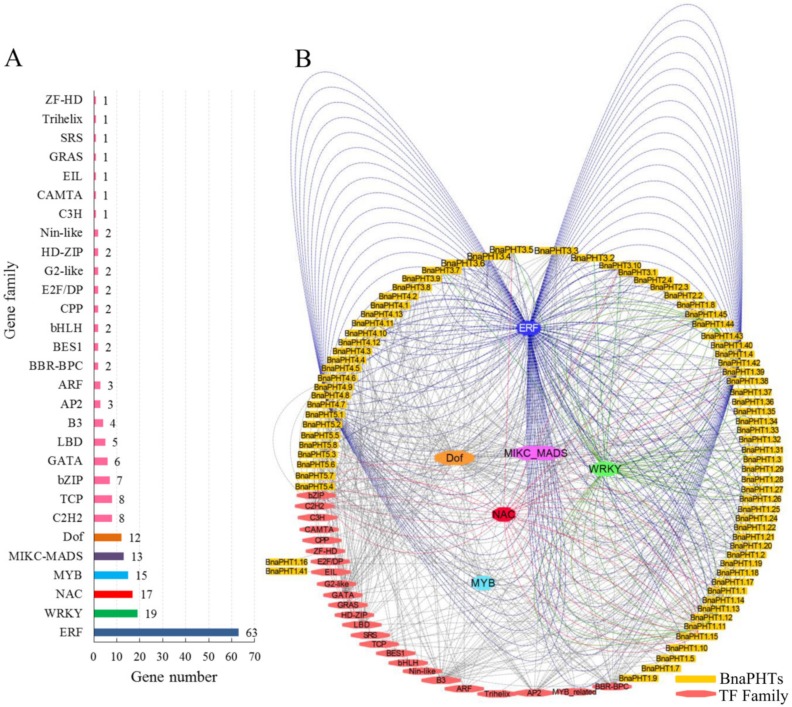
Transcription factor (TF) binding network of *BnaPHTs*. (**A**): The TF gene families that may bind to the promoter regions of *BnaPHTs*; (**B**): The TFs binding network of *BnaPHTs* based on the anlayses in PlantTFDB database.

Gene expression and even function is commonly regulated by many kinds of transcription factor genes (TFs). To explore the transcriptional regulating mechanism of *BnaPHTs*, we analyzed the putative TFs binding sites in their promoter regions (upstream 1500 bp) by PlantTFDB database, and constructed their TF-binding network using Cytoscape software (Figure 5).

In general, various putative TF binding sites were observed in the promoter regions of candidate *BnaPHTs*, belonging to 30 TF gene families (Table S4). The most enriched TFs belong to the ERF (Ethylene responsive element binding factors, 63 genes), WRKY (WRKY DNA-binding protein, 19 genes), NAC (NAM, ATAF, and CUC TFs, 17 genes), MADS-box (MCM1, AG, DEF, and SRF TFs, 13 members), MYB (myeloblastosis, 15 genes, including 12 R2R3-MYB and three MYB-related genes) and Dof (DNA binding with one finger, 12 genes) families (Figure 5A). Moreover, many TFs are likely to bind to the same target gene, such as the 63 candidate ERF TFs may target to 21 *BnaPHTs* (Figure 5B). For example, many of the candidate WRKY TFs may bind to *BnaPHT1.11* and *BnaPHT1.44* genes promoters, suggesting these two *BnaPHTs* might be co-regulated by many WRKY TFs. Similarly, *BnaPHT1.42* and *BnaPHT4.7* were found to be the common targets of many ERF TFs, while many NAC TFs tend to target *BnaPHT1.15* and *BnaPHT5.4* (Figure 5B). In contrast, the Dof and MADS-box TFs are likely to target a much wider *BnaPHTs*, where the 13 MIKC-MADS-box TFs may regulate 27 *BnaPHTs* and the 12 Dof TFs may regulate 28 *BnaPHTs* (Figure 5B and Table S3). The remaining TF families only bind to a few *BnaPHTs* promoters as well, such as C3H TF may only bind to *BnaPHT5.5* promoter, EIL TF may bind to *BnaPHT1.41* promoter, while ZF-HD TF might bind to *BnaPHT1.16* promoter (Figure 5).

To date, several TF families were reported to be involved in the transcriptional regulation of *PHTs* [34,35]. For example, the MYB-related gene *PHR1* (phosphate starvation response), regulated *PHT1* gene expression under low Pi condition through binding to the P1BS (PHR1-binding sequence) or P1BS-like domain; and the expressions of *AtPHT1;1* and *AtPHT1;4* genes were negatively regulated by *MYB62* [34]. Accordingly, our results showed that many MYB homologs may bind to *BnaPHTs* promoters, suggesting their possible roles in Pi transport. It was previously reported that *WRKY45* could positively regulate *AtPHT1;1* expression under Pi starvation by binding to two W-boxes in its promoter [35]. Consequently, we found that 19 WRKY TFs may bind to *BnaPHTs* promoters, implying possible regulating roles of this kind of TFs in *BnaPHTs* expression.

Our results indicate that the expressions of *BnaPHTs* may be regulated by many types of TFs, especially the ERF, WRKY, MYB, Dof and MADS-box family members.

### 2.7. Spatial and Temporal Expressions of BnaPHTs

Gene expression is related to its encoded protein function. So we inspected the expression patterns of the 81 candidate *BnaPHTs* in 50 *B. napus* tissues/organs across different developmental stages, based on the RNA-seq data in NCBI (BioProject ID PRJNA358784). Twenty-seven *BnaPHTs* of PHT1 family with no or weak (FPKM < 1) expression levels that might be pseudogenes or have spatially or temporally restricted expressional pattern, were excluded from the heatmap.

**Figure 6:**
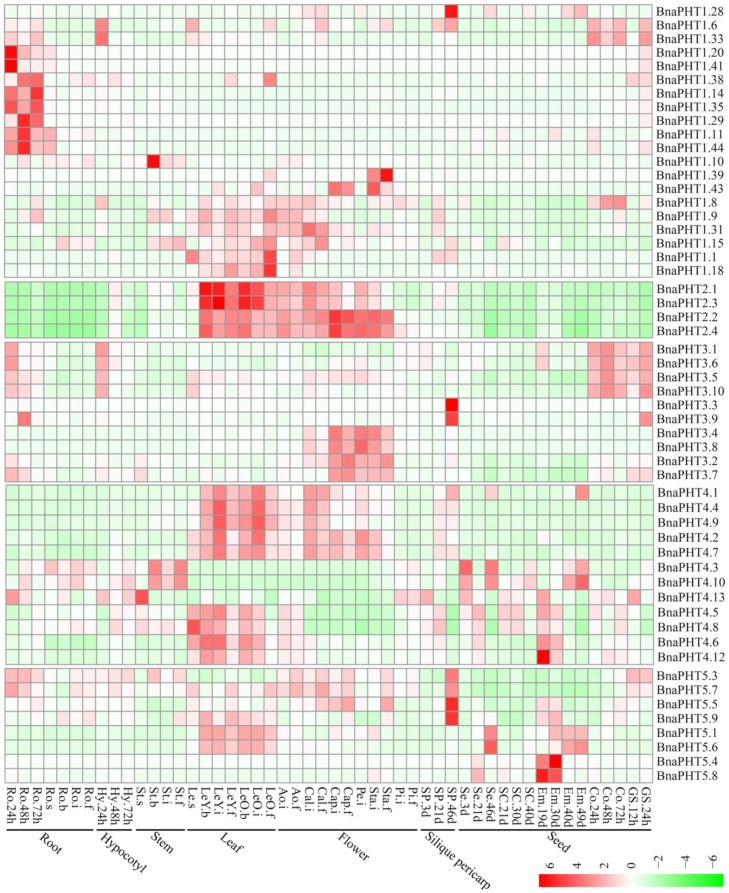
Expression pattern of *BnaPHTs* in 50 tissues during plant development. Ro = root, St = stem, Le = leaf, Sp = silique pericarp, Sc = seed coat, Em = embryo, Ao = anthocaulus, Se = seed, Hy = hypocotyl, GS =germination seeds, Cap = capillament, Pi = pistil, Cal = calyx, Co = cotyledon, Pe = petal. The “h”, “d”, “i”, “f”, “s” indicate hour, day, seeding, budding, initial flowering, and full-bloom stages, respectively. The *BnaPHTs* with no or weak expression levels (FPKM < 1) were removed from the heatmap. The color bar at the low right represents log2 expression value (FPKM ≥ 1): green represents low expression, and red represents high expression.

In general, the majority of *BnaPHTs* were partial to highly express in leaf and flower tissues (Figure 6), indicating a temporal and a spatial expression trend. Consistent with their diverse sequence characteristics, the expression profiles of *BnaPHTs* in PHT1, 3 and 4 families are relative divergent, which may explain their possible functional division in phosphorus transport process. For instance, the expression pattern of PHT1 family could be classified into three main categories: the first type is preferentially expressed in germination seeds (*BnaPHT1.28*, *BnaPHT1.6* and *BnaPHT1.33*); the second type was highly expressed in radicle (*BnaPHT1.20*, *BnaPHT1.41*, *BnaPHT1.14*, *BnaPHT1.35*, *BnaPHT1.11*, *BnaPHT1.44, BnaPHT1.38*, *BnaPHT1.29* and *BnaPHT1.10*); while the remaining are mainly expressed in leaf and flower tissues. The expression profiles of *BnaPHT3s* can also be divided in to three categories: the first category was expressed in germination seeds and cotyledons, the second was mainly expressed in silique pericarps, and the rest was highly expressed in flower tissues. The expression profiles of the *BnaPHT3s* could be related to their predicted mitochondrial localization according to all of them contained the mito-carr domain. Similarly, the expressions of *BnaPHT4s* can be divided into two major groups: the first one consists of five members (*BnaPHT4.1, BnaPHT4.4, BnaPHT4.9, BnaPHT4.2* and *BnaPHT4.7*) that were highly expressed in leaf and flower tissues, while the second has relatively wider expression levels in the tissues investigated. In contrast, the expression profiles of the other two families are more conserved than the above three ones (Figure 6). In PHT2 family, the expressions of candidates are very similar and are highly expressed in leaf and flower tissues. Combined with their subcellular location analysis results, we predicted that they maybe affect Pi allocation at chloroplast. Similarly, the *BnaPHT5s* were mainly expressed in leaf, flower and silique pericarps tissues, and some members were also highly expressed in seed and embryo tissues. The expression patterns together with their subcellular location analysis suggested that the *BnaPHT5s* may function in Pi storage in many tissues.

In general, most of the *BnaPHTs* tend to be expressed in leaf and flower tissues. Moreover, the expression patterns within each family or group are similar, implying their functional conservation.

### 2.8. Expression Analysis of BnaPHTs under Five Hormone Inductions

Plant hormones participate in the regulation of diverse plant processes. For instance, auxin is a key regulator for virtually every aspect of plant growth and development [36]; Gibberellin (GA) is a bioactive growth regulator which controls seed germination, stem elongation, flowering [37]; 6-Benzylaminopurine (6-BA) could stimulate cell division or cytokinesis [38]. Moreover, phytohormones are also known to be associated with nutrient signaling [39]. Therefore, we explored the expression patterns of *BnaPHTs* under five exogenous hormone treatments (IAA, ACC, ABA, GA_3_, and 6-BA) in *B. napus* roots, based on our RNA-Seq dataset (BioProject ID PRJNA608211). Forty-four *BnaPHTs* with no or weak expression levels (FPKM < 1) were excluded from the heatmap.

**Figure 7:**
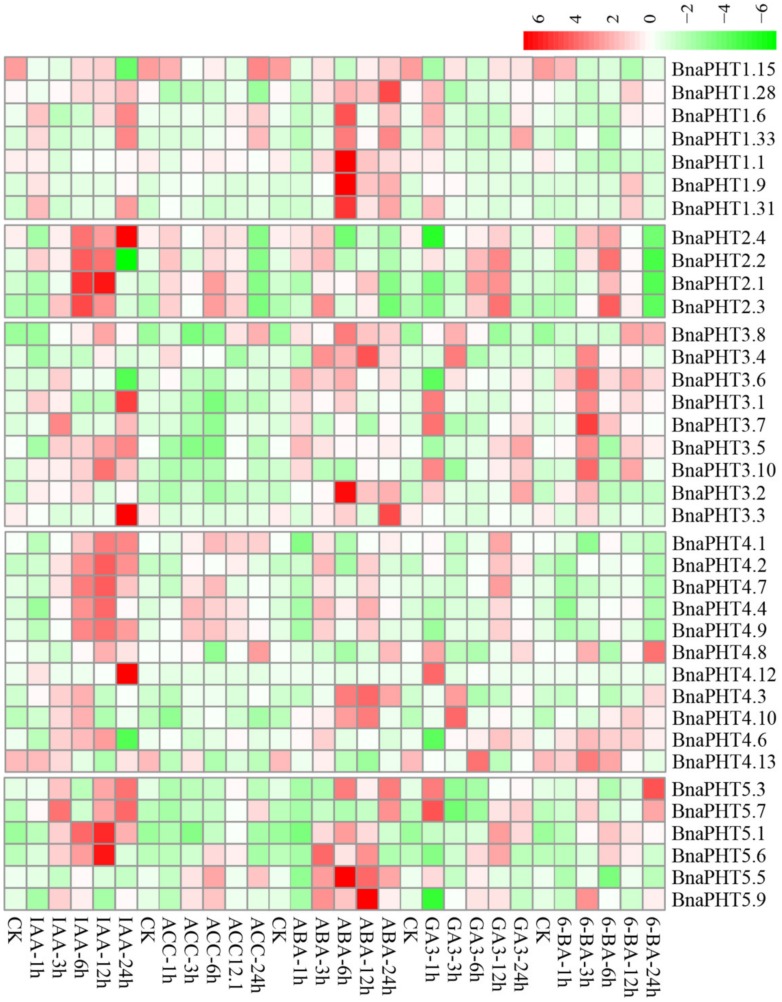
Expression profiles of *BnaPHTs* under five hormone inductions in *B. napus* seedling roots by RNA-seq. CK: no extra hormone inductions (0 h), IAA: indoleacetic acid, ACC: 1-amino cyclopropanecarboxylic acid, ABA: abscisic acid, GA_3_: gibberellin acid 3, 6-BA: cytokinin. The “1 h”, “3 h”, “6 h”, “12 h”, and “24 h” represent hours after treatment. The color bar in the upper right represents log2 expression values (FPKM ≥ 1): green represents low expression and red represents high expression.

As mentioned above, the expressions of most *BnaPHTs* were generally low in roots (Figure 6). However, the expressions of many genes in roots were induced by the five hormone treatments (Figure 7). Among the five PHT families, the *BnaPHT1s* (e.g., *BnaPHT1.1*, *BnaPHT1.6*, *BnaPHT1.9*, and *BnaPHT1.31*) were evidently up-regulated by ABA treatment; the *BnaPHT2s* (*BnaPHT2.1*-*BnaPHT2.4*) were up-regulated by all the five hormone treatments, especially IAA; the *BnaPHT3s* (*BnaPHT3.1*-*BnaPHT3.8*, and *BnaPHT3.10*) were up-regulated by IAA, ABA, GA_3_ and/or 6-BA treatments; whereas the genes in PHT5 family (*BnaPHT5.1*, *BnaPHT5.3*, *BnaPHT5.5- BnaPHT5.7*, and *BnaPHT5.9*) were evidently up-regulated by IAA and/or ABA treatments (Figure 7). Morevoer, the expressions of homologs in a same family are generally similar, such as *BnaPHT1.1*, *BnaPHT1.9* and *BnaPHT1.31* having similar expression patterns under ABA treatment; and *BnaPHT2.1*-*BnaPHT2.4* having similar expression patterns under each of the five hormone inductions (Figure 7). These results suggest that the functions of *PHTs* in the same gene family are relative conserved. Notably, the expression patterns of *BnaPHT4s* were divided into two main patterns: the first type (*BnaPHT4.1*, *BnaPHT4.2*, *BnaPHT4.4*, *BnaPHT4.7* and *BnaPHT4.9*) was obviously up-regulated by IAA treatment, while another was up-regulated by IAA, ABA, GA_3_ and/or 6-BA treatments under different trends. This suggests possibility functional divergence in the PHT4 family.

Previous reports have proven that the expressions of *PHTs* by hormone-induction are similar to that by Pi stress, suggesting there is a linkage between hormone treatment and Pi starvation [40,41,42]. For instance, in rice, *OsPHT1;8* is involved in the cross-talk between Pi and auxin signaling, providing an evidence for the linkage between hormone and -Pi response [43]. Similarly, we found that the expressions of four *BnaPHT1s* (*BnaPHT1.6*, *BnaPHT1.9*, *BnaPHT1.31* and *BnaPHT1.33*) which were up-regulated under Pi deficiency [29], were also up-regulated by IAA treatment (Figure 7). This implies a linkage between IAA treatment and Pi starvation. Moreover, cytokinin has been shown to be involved in the suppression of Pi starvation-induced gene expression [44]. Consistently, our results showed that the expressions of most *BnaPHT1s* were suppressed by 6-BA treatment (Figure 7), indicating similar mechanism may exist in *B. napus*.

In summary, our results demonstrate that exogenous hormones could strongly induce the expressions of many *BnaPHTs* in *B. napus* roots (especially IAA and ABA), indicating that hormones have an important role in their actions. Further research is required to elucidate their precise roles in hormone and −Pi signaling.

### 2.9. Expression Analysis of BnaPHTs in Response to High-Pi and Low-Pi Conditions by qRT-PCR

The transcription levels of *PHTs* are generally responsive to Pi levels. Among the five PHT families, members of PHT1 family are high-affinity Pi transporters in plants [13,14,18,45] and are up-regulated under Pi deprivation conditions [2,46]. On the basis of our spatiotemporal expression analysis (Figure 6) and the known functions of *Arabidopsis* homologs, three homologous pairs (*BnaPHT1.20*/*BnaPHT1.41*, *BnaPHT1.11*/*BnaPHT1.44* and *BnaPHT1.14*/*BnaPHT1.35*) which were highly expressed in roots were selected for further qRT-PCR assay to explore their potential roles in response to low Pi (1 μM, −Pi) and high Pi (2 mM, +Pi) conditions in *B. napus* seedling roots.

**Figure 8:**
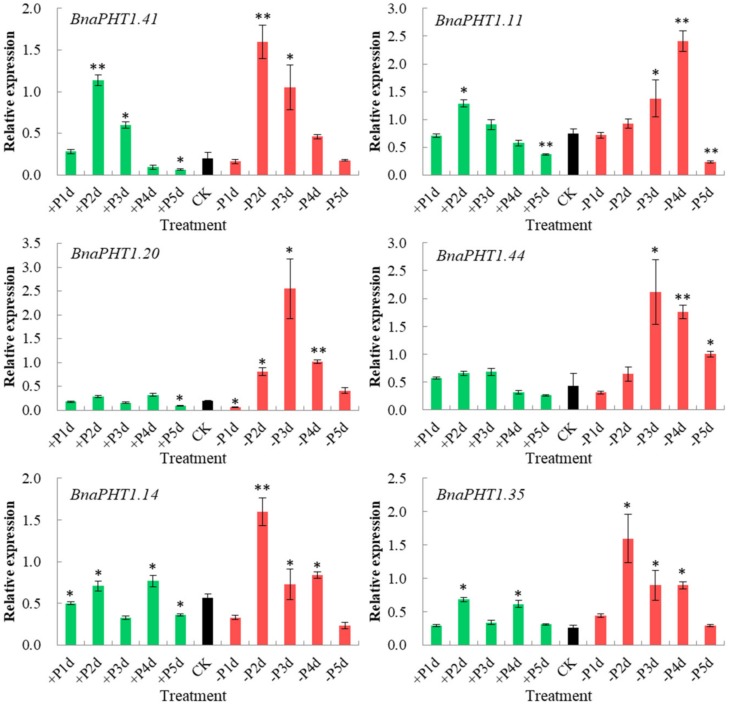
Expressions of six *BnaPHT1s* under low- or high- Pi treatments by qRT-PCR. The transcript levels were determined in *B. napus* seedling roots by qRT-PCR method under low (-Pi) or high Pi (+Pi) conditions. CK: normal Pi condition; +P1d~+P5d: +Pi condition on 1-5 day (s); -P1d~-P5d: -Pi condition on 1~5 day (s). The *B. napus Actin7* (*BnActin7*) (GenBank accession no. AF024716) was used as the reference gene. The red bar represents the expressions of *BnaPHTs* under -Pi conditions; the green bar represents the expressions of *BnaPHTs* under +Pi conditions; and the black bar represents the expressions of *BnaPHTs* under normal Pi conditions (CK). Error bars indicate the standard deviation of three independent experiments. *: Significant difference (0.05 > *p* > 0.01); **: Extremely significant difference (*p* < 0.01).

As shown in Figure 8, all of the six genes were strongly up-regulated under −Pi condition, but were relatively less induced by +Pi stress (except for *BnaPHT1.41*), indicating they were more sensitive to −Pi condition. The expression patterns of these genes can be classified into three patterns: *BnaPHT1.41* and *BnaPHT1.11* were strongly up-regulated under both of −Pi and +Pi conditions; the two homologs of *AtPHT1.9*, *BnaPHT1.14* and *BnaPHT1.35* were up-regulated by -Pi stress while were slightly up-regulated under +Pi condition; and *BnaPHT1.20* and *BnaPHT1.44* were strongly up-regulated under -Pi condition but were not induced by +Pi stress. Notably, as a sister pair, the expression profiles of *BnaPHT1.11* and *BnaPHT1.44* under −Pi and +Pi conditions were somewhat different, where the expression of *BnaPHT1.11* was gradually increased under −Pi treatment (except for on the fifth day), while *BnaPHT1.44* had the highest expression level on the third day under −Pi condition (Figure 8). This indicates functional divergence trend of sister pair genes during evolution.

Overall, our results demonstrated that the six *BnaPHTs* in PHT1 family may involve in −Pi stress response in *B. napus* seedling roots, which provided a fundament for further gene functional research.

## 3. Discussion

### 3.1. Expansion and Evolution Mechanism of BnaPHTs

In the present study, we identified 81 PHT members in the *B. napus* genome, which represents the largest PHT gene family identified in plants to date. Given *B. napus* is an allotetraploid (A_n_A_n_C_n_C_n_) evolved from a spontaneous hybridization event (allopolyploid) between *B. rapa* (A_n_A_n_) and *B. oleracea* (C_n_C_n_) about 7500 years ago [31], and that Brassicaceae species experienced a common whole genome triplication (WGT) event during evolution, it was expected that the 21 *Arabidopsis PHTs* may be expanded to ~60 genes in *B. rapa* or *B. oleracea*, and ~120 in *B. napus* genomes, respectively. However, in this study, only 44, 26, and 81 genes were identified in these three species respectively, which revealed that 59% of *BoPHTs*, 30% of *BrPHTs* and 36% of *BnaPHTs* genes were lost during evolution. This indicates that many PHT members were lost after the WGD and allopolyploid events, in line with the important gene losses that genomes suffer shortly after WGD leading to a rapid diploidization [47]. Our colinearity analyses showed that up to 65 of the 81 *BnaPHTs* have colinear relationships. Among them, ~50.8% were inherited from *B. rapa* (~33.8%) or *B. oleracea* (~17.0%) genomes. Thus, the allopolyploid is the main driving force for *PHTs* expansion in *B. napus*, and the genes from *B. rapa* tended to be retained. Moreover, we found that ~39.5% *BnaPHTs* were derived from SE (~18.5%), HE (~7.4%), and SD (~7.4%), while only ~6.2% genes were from TD event (Table S5), suggesting that the small-scale segmental duplication events were the major contributor for the large gene expansion in *B. napus* after genomic heterogeneous doubling as well. Notably, the majority of *BnaPHTs* from HE (5 of 6 pairs, ~83.3%) were homologous exchanged from A_n_- to C_n_- subgenome, which represents an obvious bias for this event. In addition, the TD events were only observed in PHT1 family (Figure 4), suggesting a trend for this kind of gene expansion event. Together, the allopolyploid was the main driving force for the rapid expansion of *BnaPHTs*, followed by SE, SD, HE and TD events.

Gene duplication events might cause gene function differentiation, such as function redundancy, neo-functionalization, non-functionalization and sub-functionalization [48]. To explore the fates of the duplicated *BnaPHTs*, we calculated the sequence similarity and identity of the full protein, gene (DNA and cDNA) and the promoter (-1500 bp) sequences of the duplicates (Table S6). For the orthologous pairs between *B. napus* and *B. rapa* (21 pairs) or *B. oleracea* (8 pairs), the sequence identities of their protein, gene and promoter sequences were on average ~88.58%, ~77.15% and ~66.75%, respectively. Similarly, for the SE colinearity-pairs, the sequence identities of the protein, gene and promoter sequences were on average ~88.93%, ~85.78% and ~73.68%, respectively. For the HE colinearity-pairs, the sequence identities of the protein, gene and promoter sequences were on average ~95.02%, ~85.97% and ~69.70%, respectively. For the SD colinearity-pairs, the sequence identities of the protein, gene and promoter sequences were on average ~91.64%, ~83.84% and ~55.68%, respectively. For the TD colinearity-pairs, their protein, gene and promoter sequence identities were on average ~90.76%, ~81.28% and ~53.18%, respectively. Overall, the average sequence identity in the promoter regions of colinearity-pairs is ~65.50%, which is much lower than that of the protein (~89.89%) and gene sequences (~80.26%), implying the differentiation of duplications tended to occur in the promoter regions, firstly. The promoter regions in the genes from SD and TD events are likely to differentiate faster than the other events, indicating that the evolution trend of newly duplicated genes occurred in the descendant (*B. napus*).

Pearson correlation coefficient analysis showed that, except for seven colinearity-pairs which had low or no detectable expression levels in most tissues in *B. napus*, ~73.3% of the remaining pairs (including SE, HE, SD and TD events) have very similar expression patterns (Pearson correlation coefficient value > 0.8), while ~26.7% pairs have different expression patterns (Pearson correlation coefficient value < 0.6) (Table S6). This indicates that most of the colinearity-pairs are functionally redundant, while a few duplicates underwent expression differentiation that may lead to functional divergence (e.g., neo-functionalization and sub-functionalization) during evolution.

### 3.2. Function and Expression Characteristics of Each PHT Family

Pi is one of the keys as well as most limiting mineral nutrients essential for plant growth and development. The *PHTs* play important roles in Pi uptake and translocation. Since the cloning of the first plant PHT1 family gene in *Arabidopsis* [49], an increasing number of *PHTs* have been identified and functionally characterized in various plant species (Table S7).

The plant *PHTs* were commonly classified into five families, designated PHT1-5. Among them, PHT1 family is the most intensively studied one which acts as high affinity Pi transporter. To date, the majority PHT1 homologs were reported to play key roles in Pi uptake and translocation. For example, *Arabidopsis AtPHT1;1* and *AtPHT1;4* were responsible for Pi acquisition in both low- and high- Pi environments [14]; Rice *OsPHT1;3* acted as a crucial factor for Pi acquisition under extremely low Pi condition [50] (Table S7). In agreement with their biological roles in Pi acquisition, many of them were generally Pi-starvation-induced in roots and/or shoots [18,45,51], e.g., eight of the nine *Arabidopsis PHT1s* (except for *Pht1;6*) [13] and 10 of the 13 rice *PHT1s* [26] were regulated in roots under Pi starvation. Similarly, in this study, we proved that six *BnaPHT1s* (*BnaPHT1.11*, *BnaPHT1.14*, *BnaPHT1.20*, *BnaPHT1.35*, *BnaPHT1.41* and *BnaPHT1.44*) were regulated by Pi starvation, implying similar functions in Pi uptake and translocation. Notably, some PHT1 family members play critical roles in arbuscular mycorrhizal symbiosis and/or symbiotic Pi uptake as well, such as *MtPT4* in *M. truncatula* [52], *OsPT11* and *OsPT13* in rice [53], *AsPT1* and *AsPT4* in *Astragalus sinicus* [54]; while some members (e.g., *AtPht1;1*, *OsPht1;8* and *PvPht1;3*) play an important role in both Pi and Arsenic (As) uptake and translocation due to the similarity of Pi and As [55,56,57] (Table S7). In addition, PHT1 homologs are also involved in many other processes, such as Pi redistribution and mobilization [50], embryo development [58] (Table S7). Together, these results demonstrated that PHT1 genes not only have major roles in Pi uptake and translocation [18,45,46,47,48,49,50,51,52,53,54,55,56,57], but also have diverse biological functions in other plant processes, which is consistent with their diverse expression profile in plants [12,13,26,49,59,60].

In contrast, relatively fewer investigations have been performed on the last four PHT families which were mainly involved in Pi distribution within subcellular compartments (Table S7). The PHT2 family is generally small in each plant species (generally one member in each species) and shares extensive homology. Members of this family that have been characterized so far are low-affinity Pi transporters and act as H^+^/Pi cotransporters in plant plastids, such as *AtPHT2;1* [20,21] and *OsPHT2;1* [61]. The PHT2 homologs were predominantly expressed in green tissue [21,62,63,64,65], such as *AtPHT2;1* [21], and *EsPHT2;1* [65]. However, some PHT2 members were expressed in roots as well, and were regulated by external Pi concentration, such as *TaPHT2;1* [64]. In *B. napus*, we identified four *BnaPHT2s* that had a conserved high expression profile in leaf and flower tissues (Figure 6). This is consistent with their known roles in chloroplasts. To date, merely a few genes of PHT3 family were functionally characterized in plants. Members of this group encode mitochondrial transporters that are involved in Pi exchange between cytoplasm and mitochondria via Pi⁄H^+^ symport or Pi⁄OH^-^ antiport activities [66], and plant development and stress response [22,27,67]. Expression analysis showed that members of this family are preferentially expressed in flower or germinating seed tissues (Figure 6), indicating their potential roles in *B. napus*. PHT4 homologs are low-affinity Pi transporters as well and act as important Pi transport in Golgi apparatus and in plastids (Table S7). Interestingly, despite the limited membership of this family in each plant species (such as the six genes in *Arabidopsis*), they have a wide range of functions in plants in addition to their roles as plastid-localized Pi transporters (Table S7). For example, in *Arabidopsis*, *AtPHT4;1* gene plays a critical role in Pi availability and basal defense [68], *AtPHT4;2* influences Pi transport, starch accumulation and leaf size [69], *AtPHT4;4* is a chloroplast-localized ascorbate transporter [70]; *AtPHT4;6* facilitates the selective transport of Pi and responses to salt tolerance [24]; in *Citrullus lanatus*, *ClPHT4;2* is correlated with chromoplast Pi transporter, flesh color development, and carotenoid content [71]. Similarly, *Citrus sinensis CsPHT4;2* could be also involved in Pi transporter and enhance carotenoid accumulation [72]. Accordingly, we found that the sequence feature and expression profile of *BnaPHT4s* were relative divergent (Figure 2 and Figure 6), indicating their diverse functions in *B. napus*. The PHT5 family (also named SPX-MFS family) was considered as vacuolar Pi transporter [16]. PHT5 genes are well known to regulate cytoplasmic Pi homeostasis, and are required for fitness and plant growth as well [16] (Table S7). For instance, *Arabidopsis AtPHT5;1* is essential for mediating vacuolar Pi storage and plant adaptation to fluctuating Pi level [16,73]; Rice *OsSPX-MFS1* and *OsSPX-MFS3* were also involved in maintaining Pi homeostasis [74,75]. On the basis of global expression analysis, we observed that many *BnaPHTs* were strongly up-regulated by exogenous hormone inductions (ABA, IAA, GA_3_, 6-BA and ACC) in *B. napus* roots (Figure 7), suggesting a potential linkage between hormone treatment and Pi signaling. Moreover, the subcellular localization of candidate *BnaPHTs* are highly consistent with the known functions of homologs in each family (Table S1), suggesting an important cue for gene functions as well.

In brief, the five PHT families have key roles in plant Pi uptake, transport and/or storage, and also have important roles in plant stress response, growth and development. Our results offer a useful basis for future research work on plant *PHTs* functions as well as the long-term goal of improving the Pi use efficiency of oil crops.

## 4. Materials and Methods

### 4.1. Identification and Phylogenetic Analyses of PHT Proteins in B. napus Genome

The sequences of the *Arabidopsis PHTs* were downloaded from TAIR (http://www.arabidopsis.org/). To identify the PHT genes in *B. napus* genome, a preliminary BLASTP [76] search was performed using the sequences of known *Arabidopsis* PHT proteins as queries, based on the available *B. napus* genome database in Genoscope (Darmor–*bzh*, http://www.genoscope.cns.fr/brassicanapus/) [31]. Only hits with E-values < 1.0 were considered as the candidate PHT gene family members. Subsequently, the candidate sequences were verified by SMART (http://smart.embl-heidelberg.de/) to ensure they own the typical domains of the five PHT families, respectively. The same methods were applied to identify the candidate *PHTs* in *B. rapa* and *B. oleracea* genomes in Phytozome v12 (https://phytozome.jgi.doe.gov/) [77], respectively.

Multiple sequence alignment of full length proteins of candidate *PHTs* was performed using the online MAFFT version 7 software under default parameters (https://mafft.cbrc.jp/alignment/server/). The result was then edited by MEGA version 7.0 software [78]. Based on the multiple sequence alignment of candidates, a neighbor-joining (NJ) tree was constructed by MEGA 7.0 with the following parameters: p-distance, pairwise deletion, and a bootstrap with 1000 replicates. The best evolutionary model of candidate PHT proteins was predicted by the Akaike information criterion (AIC) using MEGA7.0. The maximum-likelihood (ML) tree was constructed by MEGA 7.0 with 100 replicates, and JJT amino acid substitution model with estimation of the gamma distribution shape parameter (JJT + G), based on the multiple sequence alignment. The phylogenetic tree files were viewed and edited using FigTree v1.3.1 (http://tree.bio.ed.ac.uk/software/figtree/).

### 4.2. Biochemical Properties, Subcellular Localization and Sequence Analysis of BnaPHTs

The molecular weight (MW), theoretical isoelectronic points (PI), and grand average of hydropathy (GRAVY) of candidate BnaPHTs were calculated using ProtParam tool (https://web.expasy.org/protparam/) [79]. The information of cDNA and genomic sequences for candidate *BnaPHTs* were acquired from the *B. napus* genome as well. The gene structures of *BnaPHTs* (intron distribution, position, and phase in the full-length coding region) were viewed using Gene Structure Display Server (GSDS) 2.0 online software (http://gsds.cbi.pku.edu.cn/) [80]. The intron insertion information of the *PHTs* in *Arabidopsis, B. rapa* and *B. oleracea* was acquired from Phytozome v12. The information of the domains of BnaPHTs was acquired from SMART (http://smart.embl-heidelberg.de/) and Pfam (http://pfam.xfam.org/) respectively. The prediction of transmembrane helices was performed by TMHMM online software (http://www.cbs.dtu.dk/services/TMHMM-2.0/). Subcellular localization of BnaPHTs was predicted by Cell-PLoc 2.0 (http://www.csbio.sjtu.edu.cn/bioinf/Cell-PLoc-2/) [81], TargetP-2.0 (http://www.cbs.dtu.dk/services/TargetP/) and Pprowler (http://bioinf.scmb.uq.edu.au:8080/pprowler_webapp_1-2/index.jsp) respectively.

### 4.3. Chromosomal Location and Gene Duplication Analysis

The information regarding chromosome length and gene locations of candidate *BnaPHTs* were obtained from *B. napus* genome in Genoscope database as well. The collinearity relationship of *PHTs* from *Arabidopsis*, *B. napus*, *B. oleracea* and *B. rapa* was analyzed by CoGe online software (https://genomevolution.org/coge/). The duplication events were defined based on the cross-genome collinearity analysis of candidate *PHT*s (orthologous gene pairs in orthologous blocks). The closely related *PHT*s that were physically located to each other on a given chromosome and no more than one gene intervention were defined as tandemly duplicated genes. The Mapchart software was used to draw the chromosome map of the *BnaPHTs.*

### 4.4. Transcription Factor (TF) -Binding Network Analysis

The putative transcription factor-binding site analysis was performed by PlantTFDB database (http://planttfdb.cbi.pku.edu.cn/prediction.php) using the promoter sequences (upstream 1500 bp) of *PHTs*. The corresponding TF-binding network was visualized by Cytoscape [82] software.

### 4.5. Development and Hormone-Induced Expression Profile Analysis of BnaPHTs

The temporal and spatial expression profiles of *BnaPHTs* in 50 different tissues/organs (root, stem, leaf, flower, seed, and silique tissues) of *B. napus* cultivar ‘Zhongshuang 11′ (ZS11) at different developmental stages (seed germination, seedling, budding, initial flowering, full-bloom and seed maturation) were analyzed based on the RNA-seq data in NCBI (BioProject ID PRJNA358784). Similarly, the expression patterns of *BnaPHTs* under five major hormone inductions (IAA, GA_3_, 6-BA, ABA and ACC) in *B. napus* ZS11 seedling roots were evaluated based on our recently constructed RNA-seq dataset (BioProject ID PRJNA608211). Genes with no or weak expression value (FPKM<1) were removed from further analyses. The RNA-Seq data (FPKM ≥ 1) was log2-transformed, and the heatmap was performed using R package [83].

### 4.6. Plant Materials and Growth Condition

Seeds of ZS11 were obtained from the College of Agriculture and Biotechnology, Southwest University. The seeds were germinated in individual plastic pots filled with sand, and grown in an artificial climatic chamber at 22 °C with a 16:8 h photoperiod (day:night). The seedlings at five-leaf stage were used for low−Pi (−Pi) and high−Pi (+Pi) treatments, respectively. For each treatment, three biological replicates were performed, and each replicate contained five plants. The seedlings were treated in 1/2-strength Hoagland solution supplemented with 1 mM KH_2_PO_4_ (Control, CK), 1/2-strength Hoagland solution with 1 μM KH_2_PO_4_ (-Pi treatment), and 1/2-strength Hoagland solution with 2 mM KH_2_PO_4_ (+Pi treatment). The root tissues were harvested on 1, 2, 3, 4 and 5 days after the treatments, and were immediately frozen in liquid nitrogen and then stored at −80 °C for RNA isolation.

### 4.7. Real-Time PCR Analyses of BnaPHTs under Low- and High- Pi Treatments

Total RNA of each sample was extracted using EASYspin total RNA Extraction kit (Biomed, Beijing). The quality and concentration of the total RNA was determined using gel electrophoresis and a NanoDrop 2000 spectrophotometer, to ensure it met the criteria of A260/280 ratio = 1.8-2.1 and A260/230 ratio ≥ 2.0. The RNA sample was treated with DNase I (Promega, USA), and then was used for first-strand cDNA synthesis in a 20 μL reaction volume according to the manufacturer’s instructions of M-MuLV RT kit (Takara Biotechnology, Japan). The primers used in this analysis are listed in Table S8. *B. napus Actin7* gene (*BnActin7*) (GenBank accession no. AF024716) was used as the reference gene. The SYBR-Green PrimeScript RT-PCR Kit (Takara Biotechnology, Japan) was used for real-time PCR analyses by the CFX Connect™ Real-Time System (Bio-Rad, Chongqing, China). The thermocycling parameters were as follows: an initial denaturation for 3 min at 95 °C, followed by 45 cycles of a denaturation step at 95 °C for 15 s, and an annealing step at 58 °C for 20 s. The fluorescence was measured after the extension step. Three biological replicates were applied for each treatment, and each treatment consisted of three technical replicates. Expression levels were calculated as the mean signal intensity across the three replicates. The relative expression levels of *BnaPHTs* were determined using the 2(^−ΔΔCt^) method [84]. Data are the mean ± standard deviation of three independent experiments. Error bars represent the standard errors from three independent experiments. Expression difference in *BnaPHTs* following Pi treatments was assessed by One-way ANOVA test (*p < 0.05; **p < 0.01) using Excel 2010.

## Supplementary Materials

Supplementary materials can be found at https://www.mdpi.com/1422-0067/21/5/1752/s1. Table S1: Features of the 81 PHT genes identified in *Brassica napus* (*BnaPHTs*). Table S2: Features of the PHT genes (*PHTs*) in *B. napus*, *Arabidopsis*, *Brassica rapa* and *Brassica oleracea.* Table S3: Protein domains of PHT proteins in *B. napus.* Table S4: Transcription factor (TF) binding sites in the promoter regions of *BnaPHTs.* Table S5: Duplication events of *BnaPHTs.* Table S6: Homology and expression correlation analysis of 51 sister pairs of *BnaPHTs.* Table S7: The functionally characterized plant *PHTs.* Table S8: List of the primers used for the Real-time PCR analyses. Figure S1: The Maximum Likelihood (ML) trees of the candidate PHT proteins from *Brassica napus*, *Arabidopsis*, *Brassica rapa* and *Brassica oleracea*. Figure S2: The intron pattern of the five PHT families in *B. napus*, *Arabidopsis*, *B. rapa* and *B. oleracea.*
