# Supplementary material for: Global Survey and Expressions of the Phosphate Transporter Gene Families in Brassica napus and Their Roles in Phosphorus Response
Source: Int J Mol Sci. 2020 Mar 4;21(5):1752. doi: 10.3390/ijms21051752 (PMC7084545; doi:10.3390/ijms21051752)
Supplement: Supplementary file 1 [file ijms-21-01752-s001.zip › ijms-724326-supplementary/Figure S1.pdf]

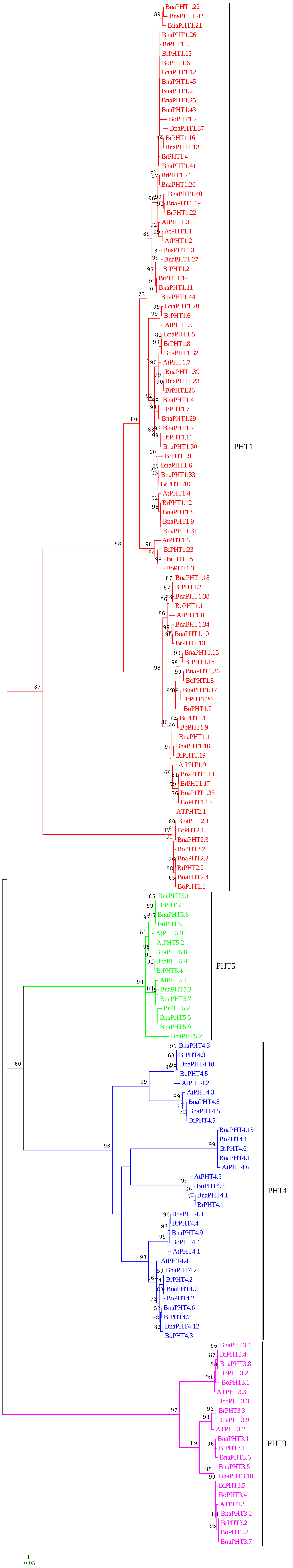
BnaPHT1.22
BnaPHT1.42
BnaPHT1.21
BnaPHT1.26
BrPHT1.3
BrPHT1.15
BoPHT1.6
BnaPHT1.12
BnaPHT1.45
BnaPHT1.2
BnaPHT1.25
BnaPHT1.43
BoPHT1.2
BnaPHT1.37
BrPHT1.16
BnaPHT1.13
BrPHT1.4
BnaPHT1.41
BrPHT1.24
BnaPHT1.20
BnaPHT1.40
BnaPHT1.19
BrPHT1.22
AtPHT1.3
AtPHT1.1
AtPHT1.2
BnaPHT1.3
BnaPHT1.27
BrPHT1.2
BrPHT1.14
BnaPHT1.11
BnaPHT1.44
BnaPHT1.28
BrPHT1.6
AtPHT1.5
BnaPHT1.5
BrPHT1.8
BnaPHT1.32
AtPHT1.7
BnaPHT1.39
BnaPHT1.23
BrPHT1.26
BnaPHT1.4
BrPHT1.7
BnaPHT1.29
BnaPHT1.7
BrPHT1.11
BnaPHT1.30
BrPHT1.9
BnaPHT1.6
BnaPHT1.33
BrPHT1.10
AtPHT1.4
BrPHT1.12
BnaPHT1.8
BnaPHT1.9
BnaPHT1.31
AtPHT1.6
BrPHT1.23
BrPHT1.5
BoPHT1.3
BnaPHT1.18
BrPHT1.21
BnaPHT1.38
BoPHT1.1
AtPHT1.8
BnaPHT1.34
BnaPHT1.10
BrPHT1.13
BnaPHT1.15
BrPHT1.18
BnaPHT1.36
BoPHT1.8
BnaPHT1.17
BrPHT1.20
BoPHT1.7
BrPHT1.1
BoPHT1.9
BnaPHT1.1
BnaPHT1.16
BrPHT1.19
AtPHT1.9
BnaPHT1.14
BrPHT1.17
BnaPHT1.35
BoPHT1.10
ATPHT2.1
BnaPHT2.1
BrPHT2.1
BnaPHT2.3
BoPHT2.2
BnaPHT2.2
BrPHT2.2
BnaPHT2.4
BoPHT2.1
PHT1
BnaPHT5.1
BrPHT5.1
BnaPHT5.6
BoPHT5.1
AtPHT5.3
AtPHT5.2
BnaPHT5.8
BnaPHT5.4
BrPHT5.4
AtPHT5.1
BnaPHT5.3
BnaPHT5.7
BrPHT5.2
BnaPHT5.5
BnaPHT5.9
BnaPHT5.2
PHT5
BnaPHT4.3
BrPHT4.3
BnaPHT4.10
BoPHT4.5
AtPHT4.2
AtPHT4.3
BnaPHT4.8
BnaPHT4.5
BrPHT4.5
BnaPHT4.13
BoPHT4.1
BrPHT4.6
BnaPHT4.11
AtPHT4.6
AtPHT4.5
BoPHT4.6
BnaPHT4.1
BrPHT4.1
BnaPHT4.4
BrPHT4.4
BnaPHT4.9
BoPHT4.4
AtPHT4.1
AtPHT4.4
BnaPHT4.2
BrPHT4.2
BnaPHT4.7
BoPHT4.2
BnaPHT4.6
BrPHT4.7
BnaPHT4.12
BoPHT4.3
PHT4
BnaPHT3.4
BrPHT3.4
BnaPHT3.8
BoPHT3.2
BoPHT3.1
ATPHT3.3
BnaPHT3.3
BrPHT3.3
BnaPHT3.9
ATPHT3.2
BnaPHT3.1
BrPHT3.1
BnaPHT3.6
BnaPHT3.5
BnaPHT3.10
BrPHT3.5
BoPHT3.4
ATPHT3.1
BnaPHT3.2
BrPHT3.2
BoPHT3.3
BnaPHT3.7
PHT3
0.05

Figure S1. The Maximum Likelihood (ML) tree of the candidate PHT proteins from *Brassica napus*, *Arabidopsis*, *Brassica rapa* and *Brassica oleracea*. The phylogenetic tree was constructed using the 165 candidate PHT proteins in *Arabidopsis* (At: 22), *B. rapa* (Br: 42), *B. oleracea* (Bo: 21), and *B. napus* (Bn: 80) which was classified into five families (PHT1-5) based on the bootstrap value and topology. Bootstrap values are indicated as percentages along the branches in the ML analysis.
