# Supplementary material for: Global Survey and Expressions of the Phosphate Transporter Gene Families in Brassica napus and Their Roles in Phosphorus Response
Source: Int J Mol Sci. 2020 Mar 4;21(5):1752. doi: 10.3390/ijms21051752 (PMC7084545; doi:10.3390/ijms21051752)
Supplement: Supplementary file 1 [file ijms-21-01752-s001.zip › ijms-724326-supplementary/Figure S2.pdf]

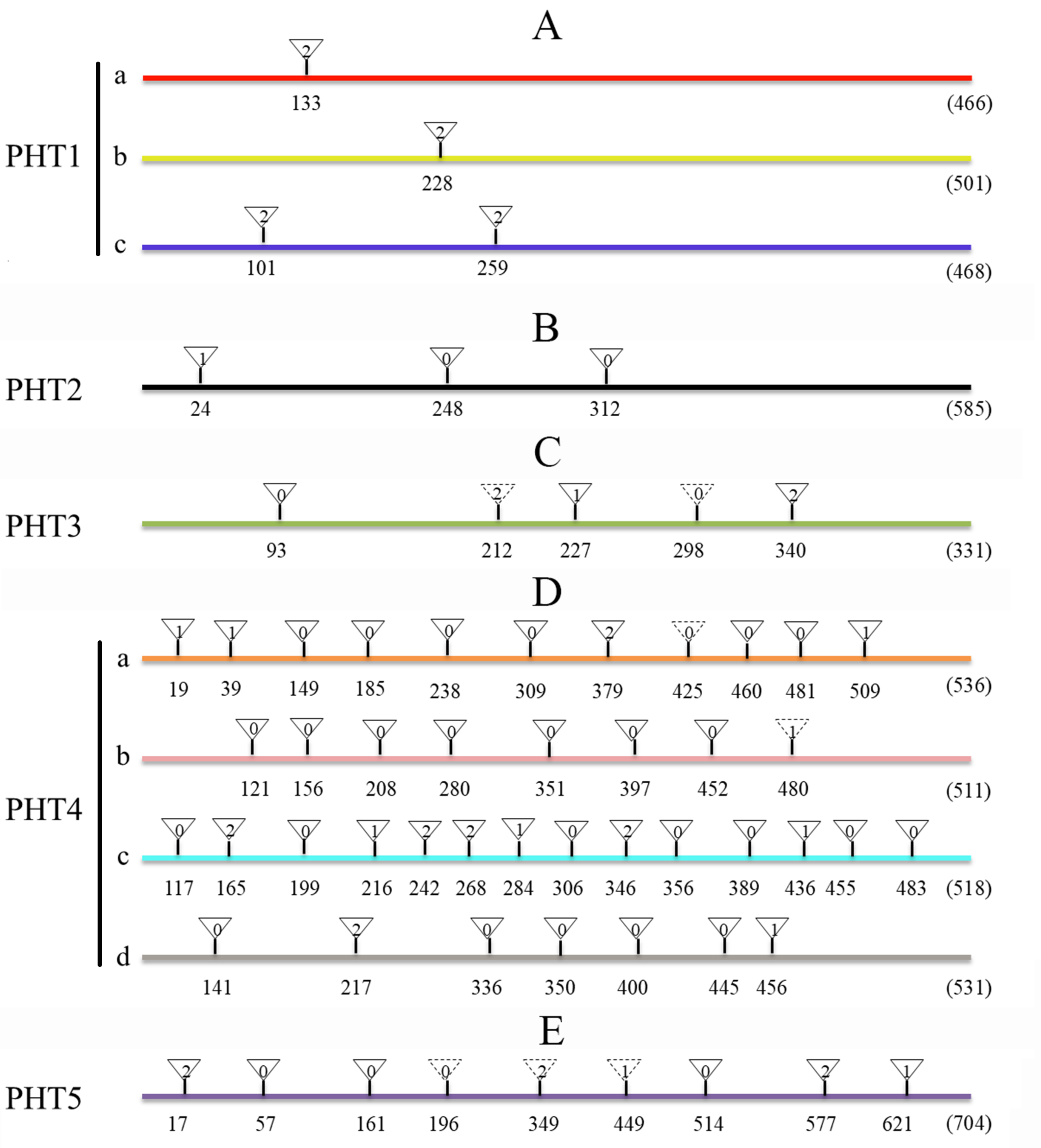

At	Br	Bo	Bn
3	8	2	12
2	7	4	12
1	2	0	1
1	2	2	4
3	5	4	9
1	2	2	4
1	1	1	2
1	1	1	1
2	2	1	4
3	2	1	8

Figure S2: The intron pattern of the five PHT families in *B. napus*, *Arabidopsis*, *B. rapa* and *B. oleracea*. Schematic diagram of intron distribution patterns of candidate PHT genes (*PHTs*). A total of ten intron patterns were identified. Intron locations are indicated by white triangles, and the number within each triangle indicates the splicing phases: 0 refers to phase 0; 1 to phase 1; and 2 to phase 2. The number of *PHTs* within each pattern of the four species is presented on the right. The correlation of intron distribution patterns and phylogenetic families is provided in Fig 2.
